# Shoulder Dislocation Reduction Using Inhaled Nitrous Oxide in the Emergency Department: Retrospective Case Series

**DOI:** 10.5811/westjem.64014

**Published:** 2026-10-26

**Authors:** Katherine Vlasica, Steven M. Hochman, Eric Steinberg, Amanda D. Russo, Jessica Carlo, Bhargavi K. Patel

**Affiliations:** * Hackensack Meridian School of Medicine, Department of Emergency Medicine, Nutley, New Jersey; † Vassar Brothers Medical Center, Department of Emergency Medicine Poughkeepsie, New York; ‡ St. Joseph’s University Medical Center, Department of Emergency Medicine, Paterson, New Jersey

## Abstract

**Introduction:**

Inhaled nitrous oxide (iNO) offers rapid onset analgesia with a favorable safety profile yet remains underused for shoulder dislocation reductions in the emergency department (ED). The objective of this study was to evaluate the use and outcomes of iNO for shoulder reduction in the ED, including whether intravenous (IV) access was required, whether successful reduction could be accomplished without additional analgesic or sedative medications under procedural sedation, and the rate of adverse effects from iNO.

**Methods:**

We conducted a retrospective chart review of shoulder dislocation reductions using iNO at two high-volume EDs over three years (2019–2022). Data collected included age, sex, indication, procedural success, IV placement, medications administered, and adverse events and was stored in a password-protected database. The primary endpoint was successful reduction with iNO as the primary procedural analgesic; secondary endpoint was occurrence of any adverse events.

**Results:**

Of 138 patients, 125 (90.6%; 95% CI, 0.84–0.95) achieved successful reduction with iNO alone without escalation to procedural sedation; 102 patients (74%) did not require IV access. Adverse events were rare (2/138 or 1.4% [95% CI, 0.02–0.05]): one patient experienced transient agitation (a known side effect of N-methyl-D-aspartate antagonism), and one had vomiting. Both were self-limited without hemodynamic changes.

**Conclusion:**

This retrospective case series, the largest of its kind to date examining iNO for shoulder dislocation reduction, demonstrated a 90.6% success rate with minimal adverse events and reduced resource utilization compared to procedural sedation. These findings support further prospective investigation of iNO for shoulder dislocation as well as other orthopedic reductions and painful ED procedures.

## INTRODUCTION

Inhaled nitrous oxide (iNO), commonly known as “laughing gas,” has been in clinical use for more than 150 years. Discovered in 1772, it took nearly 100 years for its incorporation into clinical practice. The dental profession was an early adopter after Dr. Horace Wells observed that a patient injured during a laughing gas demonstration seemed unaware of his pain until the effects of the gas wore off. The following day, Dr. Wells underwent a molar extraction under iNO anesthesia, after which he began to use iNO when performing dental procedures. In 1864, the American Dental Association recognized him as the discoverer of iNO as an analgesic.^1^ In addition to its applications in dentistry, iNO became a valuable adjunct for general anesthesia and procedural sedation due to its analgesic, anesthetic, and anxiolytic properties. Its mechanism of action involves multiple pathways. Analgesia is mediated by supraspinal opioid release in the periaqueductal gray matter, and the anesthetic and anxiolytic effects are attributed to N-methyl-D-aspartate (NMDA) receptor antagonism.^2^–^4^

Limited evidence suggests that iNO is a viable analgesic option in emergency settings.^5^–^8^ Its benefits include noninvasive administration, simple titration, and rapid onset and offset. The American Society of Anesthesiologists and American Academy of Pediatrics classify nitrous oxide at concentrations ≤ 50% as minimal sedation, excluding it from moderate procedural sedation monitoring requirements.^9^ Importantly, multiple large studies have demonstrated no clinically significant difference between 50% and 70% nitrous oxide in either sedation depth or adverse event profiles.^10^–^12^ Even at concentrations up to 70%, iNO still preserves hemodynamic status, spontaneous respirations, and protective airway reflexes.^13^ In large prospective studies, no serious adverse events—including desaturation, apnea, bradycardia, or hypotension—have been reported with nitrous oxide at concentrations up to 70%.^11^, ^14^, ^15^

Our institution has used iNO at concentrations up to 70% for over a decade. Despite the excellent safety profile of nitrous oxide at these concentrations^10^, ^11^, ^14^ and data showing that most patients remain minimally sedated even above 50%,^10^ our institutional policy requires continuous pulse oximetry and clinical observation of the patient. Traditional intravenous (IV)-based procedural sedation carries well-documented risks, including hypoxia (40–79 per 1,000 sedations), apnea (31/1,000), hypotension (15–28/1,000), and rare but life-threatening events, such as laryngospasm, aspiration, and intubation.^16^ Despite its safety, ease of use, and effectiveness, iNO has limitations. Common side effects include nausea, vomiting, dizziness, and euphoria. Due to its high solubility (35 times greater than nitrogen), iNO is contraindicated in conditions involving air-filled spaces, including pneumothorax, bullous lung disease, bowel obstruction, middle ear effusion, recent vitreoretinal surgeries, and decompression sickness.^17^

Inhaled nitrous oxide is administered via a valve mask in a mixture with oxygen. The maximum percentage of iNO with paired oxygen is 70%. However, this can be adjusted based on a patient’s tolerance and oxygen saturation. Once the procedure is completed, the iNO concentration is lowered to 0%, allowing the patient to breathe 100% oxygen for one to two minutes. This prevents postdiffusion hypoxia as the absorbed nitrous oxide from the circulation is eliminated into the alveoli, potentially causing transient dilution of alveolar oxygen.^18^

We assembled a case series to study the efficacy and potential adverse effects of using iNO as an alternative to traditional procedural sedation for shoulder reductions in a busy urban ED.

## METHODS

We searched our electronic health record (Cerner FirstNet, Oracle Health, Kansas City, MO) for all patients diagnosed with acute shoulder dislocation between January 1, 2019, and January 1, 2022, who had been treated with iNO as part of the shoulder reduction procedure. The study was conducted in the emergency departments (ED) of a high-volume urban trauma center and an affiliated community hospital. We followed the methods of the Carey and Boden 2003 critical guide. 19 Our hypothesis was that patients could have a successful reduction of a shoulder dislocation with iNO used as a single agent in performing the procedure. Inclusion criteria included patients of any age who had been diagnosed with acute shoulder dislocation and underwent a shoulder reduction procedure performed with the use of iNO.

**Population Health Research Capsule**

What do we already know about this issue?

*Existing evidence for use of inhaled nitrous oxide (iNO) in shoulder reductions in the ED is limited.*

What was the research question?

*Is iNO safe and effective for shoulder dislocation reductions in the ED?*

What was the major finding of the study?

*Shoulder reduction was successful in 125 of 138 patients (90.6%; 95% CI, 0.84–0.95) using iNO alone.*

How does this improve population health?

*Use of inhaled nitrous oxide may reduce need for procedural sedation with intravenous medication, shorten ED length of stay, and improve patient safety and resource utilization.*

Patients were excluded if they had a contraindication to iNO (pneumothorax, bowel obstruction, high suspicion for otitis media, severe chronic obstructive pulmonary disease, first or second trimester pregnancy, altered mental status), or if they declined its use. Patients were not excluded if they were given other medications for pain prior to the procedure. Abstractors were not blinded to the hypothesis of the study. Data collected included other medications given, whether an IV line had been placed, whether procedural sedation with other agents was used, successful reduction, any adverse effects, and final disposition, as well as general demographic information. Data were entered into a password-protected database only accessible to study personnel. The primary endpoint was successful reduction of the joint dislocation with the use of iNO as the primary procedural analgesic, without escalation to full procedural sedation. The secondary endpoint was the occurrence of any adverse reaction after administration of iNO. We assessed both endpoints based on documentation by the treating physician.

Inhaled nitrous oxide was administered via a Porter MXR-3000 Flowmeter device (Porter Instrument, Hatfield, PA), which delivers a continuous-flow, titratable nitrous oxide and oxygen mixture through a mask with a fail-safe mechanism preventing delivery of less than 30% oxygen. The iNO was initiated at 10% and titrated upward to a maximum of 70%. The target concentration was individualized, defined as the lowest concentration at which the patient no longer adversely responded to procedural manipulation while maintaining verbal responsiveness and intact protective reflexes. Continuous pulse oximetry and verbal responsiveness were monitored throughout the procedure. Upon completion of the reduction, iNO was discontinued, and 100% oxygen administered for five minutes to prevent diffusion hypoxia. The iNO was administered by ED staff trained in nitrous oxide delivery, with the treating physician present at bedside throughout the procedure.

This study was granted an exemption by the institutional review board at our institution. There was no outside funding source.

## RESULTS

In total, 138 patients were included in the case series, all of whom had successful shoulder reduction and were subsequently discharged from the ED. The majority, 103 (74.6%), were male; the remainder, 35 (25.4%), were female. The median age was 32.5 years, and seven patients (5%), were <18 years of age. The youngest patient was 14 years of age.

Of the 138 patients enrolled, 125 (90.6%; 95% CI, 0.84–0.95) met the primary endpoint and had successful reduction with iNO as the primary analgesic and sedative agent. The remaining 13 patients (9.4%; 95% CI, 0.05–0.16) required escalation to procedural sedation for successful reduction. The median age of the patients requiring escalation was 60 years. The most common reasons were that the patient was unable to tolerate iNO or they were inadequately sedated or pain-controlled with iNO. Eleven of the 13 received propofol; a few received ketamine or etomidate, with or without propofol. All seven pediatric patients underwent successful shoulder reductions with iNO and without escalation to procedural sedation. None had any reported side effects.

Although most subjects did not require procedural sedation, it is important to note that as with most patients coming to the ED in severe pain, many of these subjects had been medicated prior to reduction attempts while awaiting full evaluation and imaging. Of the 138 patients, 36 (26.1%) had an IV catheter placed, and 110 patients (79.7%) received at least one medication prior to reduction using iNO.

When parenteral opioids or benzodiazepines had been administered, iNO was not initiated until the clinical effects of these agents had dissipated. Of the 28 subjects who received no premedication prior to iNO, 10.7% required escalation to procedural sedation.

Of the total 138 patients, two (1.4%; 95% CI, 0.002–0.051) experienced an adverse event. One patient experienced hallucinations and agitation, a known side effect of NMDA antagonist agents like iNO and was subsequently escalated to moderate sedation. The second patient experienced nausea and episodes of vomiting, also prompting escalation. In both patients, symptoms were transient and resolved with appropriate management in the ED. No patients had any significant alteration in vital signs, cardiovascular collapse, or need for airway intervention, and none required orthopedic intervention in the ED.

## DISCUSSION

This case series, the largest of its kind in the medical literature, provides evidence of the safety and efficacy of iNO for shoulder dislocation reduction in the ED. More than 90% of the patients in our study had shoulder dislocations successfully reduced with iNO as the primary analgesic and sedative agent. To our knowledge, there is only one prior study of 45 patients from 27 years ago examining iNO for shoulder reductions, which found a success rate of 80.9% compared with our 90.6%.^20^ Intravenous medication-based procedural sedation also has an approximately 90% success rate when using IV ketamine and propofol, as found by Kruggel et al.^21^ However, the ease of use of iNO, its rapid onset and offset, and the rare occurrence of side effects (1.4% of patients in our case series, without vital sign compromise) make this agent a highly attractive option for procedural analgesia in the ED.

Notably, only 26% of patients in our study required IV placement, and among those requiring IV access, the majority received analgesia rather than procedural sedation through the IV (Figure). The reduced reliance on IV access potentially makes iNO useful in settings where IV access and cardiac monitoring are limited, such as urgent care centers and prehospital settings. While iNO has been a more widely used alternative for moderate sedation in the pediatric ED, there is less evidence in the literature regarding its use in adults.^8^, ^22^ Herres et al conducted a prospective observational study with adult ED patients, finding significant reduction in mean pain scores using iNO in the treatment of abscesses and orthopedic injuries.^23^ Similarly, Xing et al conducted a systematic review assessing iNO efficacy for analgesia in adults in the ED and found it was better than placebo, although patients experienced more dizziness and vomiting.^24^ There is currently one ongoing multicenter randomized controlled trial (NCT05388942, planned *N* = 126) specifically evaluating nitrous oxide for anterior shoulder dislocation reduction in the ED.^25^ Prior nitrous oxide data come from mixed-procedure ED analgesia studies that do not report shoulder dislocations as a distinct subgroup.

Our findings suggest that iNO is an effective alternative for analgesia and sedation during this common ED orthopedic procedure. It is a safe option with decreased need for close monitoring, compared with other commonly used medications, such as opioids, benzodiazepines, ketamine, and propofol. Importantly, concerns regarding cardiac arrhythmia and QT prolongation with high concentrations of iNO are not well supported. QT prolongation has been associated with volatile anesthetic agents such as isoflurane, sevoflurane, and desflurane, but not with nitrous oxide.^26^ Hashimoto et al demonstrated that 80% nitrous oxide did not significantly prolong the QT interval in a canine model, in contrast to halothane, thiopental, and fentanyl, and the ENIGMA-II trial (*N* = 7,112) found no increased cardiovascular complications with nitrous oxide use.^27^, ^28^ These data further support the favorable safety profile of iNO and its suitability for procedural use in settings where advanced cardiac monitoring may not be readily available.

## LIMITATIONS

This study had several limitations. Because our case-series study did not have a comparison group, we could not establish either superiority or noninferiority of iNO compared with other modalities, including traditional procedural sedation. Second, our study was conducted in a single health system, with an established practice of iNO use; therefore, our results may not be reproducible at other institutions. Third, because this was a retrospective study, our data relied on the charting of individual physicians, containing variable levels of detail as to the specifics of the procedure, such as duration of iNO administration, number of attempts, response to iNO, clinical course, and any adverse effects.[Table t1-wjem-27-1617]

Fourth, given the retrospective nature of the study, we could not control for variability in preprocedural medications, which included oral analgesics, intra-articular lidocaine, parenteral opioids, and benzodiazepines. Although opioids and benzodiazepines were allowed to clinically dissipate prior to iNO initiation, residual effects may have contributed to analgesia or anxiolysis during the reduction. This heterogeneity reflects real-world ED practice but limits the ability to attribute procedural success to iNO alone. Finally, iNO concentrations were not consistently recorded; different physicians might have used varying concentrations of iNO and oxygen.

A prospective randomized controlled trial comparing iNO to IV-based procedural sedation is warranted. Key outcome measures should include pre- and post-procedure pain scores, ED length of stay, duration of procedure, reduction success, number of attempts, and standardized protocols for medication administration and data collection. Such a study could address the limitations of the current evidence and further define the role of iNO in orthopedic procedures and other common painful ED interventions, such as abscess drainage, laceration repair, lumbar puncture, and arthrocentesis.

### Additional Considerations

Occupational waste gas exposure is a perceived barrier due to reproductive concerns for pregnant staff. Early, pre-scavenging studies linked chronic anesthetic exposure to spontaneous abortion,^29^ but these studies were limited by halogenated co-exposures, unquantified iNO levels, and absent scavenging. Modern evidence indicates no association between iNO and teratogenicity, reproductive toxicity, congenital malformation,^30^ reduced fecundability, or miscarriage when scavenging is used.^31^, ^32^ Active scavenging and ventilation are essential, and contemporary demand-valve systems with active scavenging maintain ambient iNO well below the National Institute for Occupational Safety and Health (NIOSH) 25 ppm limit.^33^, ^34^ Additionally, brief ED procedural exposures (eg, shoulder reductions) carry substantially lower risk than prolonged operating room or dental exposures.

## CONCLUSION

This case series supports the use of inhaled nitrous oxide for shoulder dislocation reductions, as a safe and effective procedural analgesia. Although we were unable to establish the superiority of iNO over traditional methods of procedural sedation, our findings suggest that the advantages of this inhaled gas could potentially extend well beyond this procedure, offering a high benefit-risk ratio that makes it an attractive option for the ED. Further prospective studies would be valuable in evaluating iNO as monotherapy for shoulder reductions, as well as a variety of other painful ED procedures. Practical limitations of iNO include logistical barriers, such as equipment availability and maintenance, staff training, and the environmental impact of nitrous oxide as a potent greenhouse gas, all of which may affect widespread adoption.

## Supplementary Information



## Figures and Tables

**Figure:**
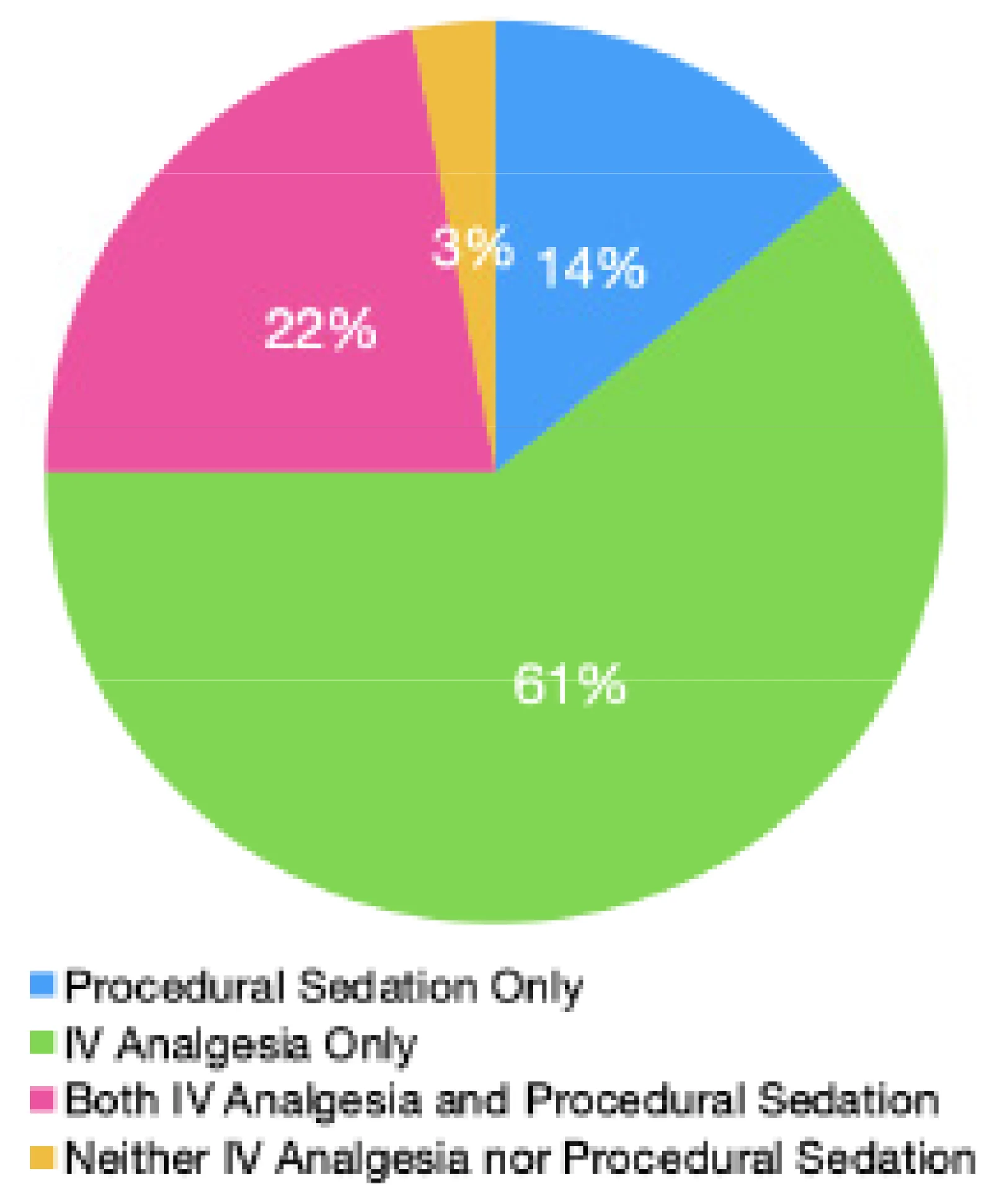
Indications for intravenous line placement, by percentage, among patients requiring access. Data are presented for the subset of patients who required IV catheterization during shoulder dislocation reduction using inhaled nitrous oxide. *IV*, intravenous.

**Table t1-wjem-27-1617:** Medications administered prior to shoulder reduction procedure with inhaled nitrous oxide.

Medication	Route	*n*
Ibuprofen	Oral	25
Acetaminophen	Oral	31
Diazepam	Oral	6
Hydromorphone	Oral	1
Ketorolac	Intramuscular	50
Midazolam	Intramuscular	1
Morphine	Intravenous	11
Fentanyl	Intravenous or intranasal	21
Intra-articular anesthetic (ropivacaine or lidocaine)	Intra-articular injection	11
