# Supplementary material for: Shoulder Dislocation Reduction Using Inhaled Nitrous Oxide in the Emergency Department: Retrospective Case Series
Source: West J Emerg Med. 2026 Oct 26;27(6):1617–22. doi: 10.5811/westjem.64014 (PMC13761820; doi:10.5811/westjem.64014)
Supplement: Supplementary file 1 [file wjem-27-1617-g001.pdf]

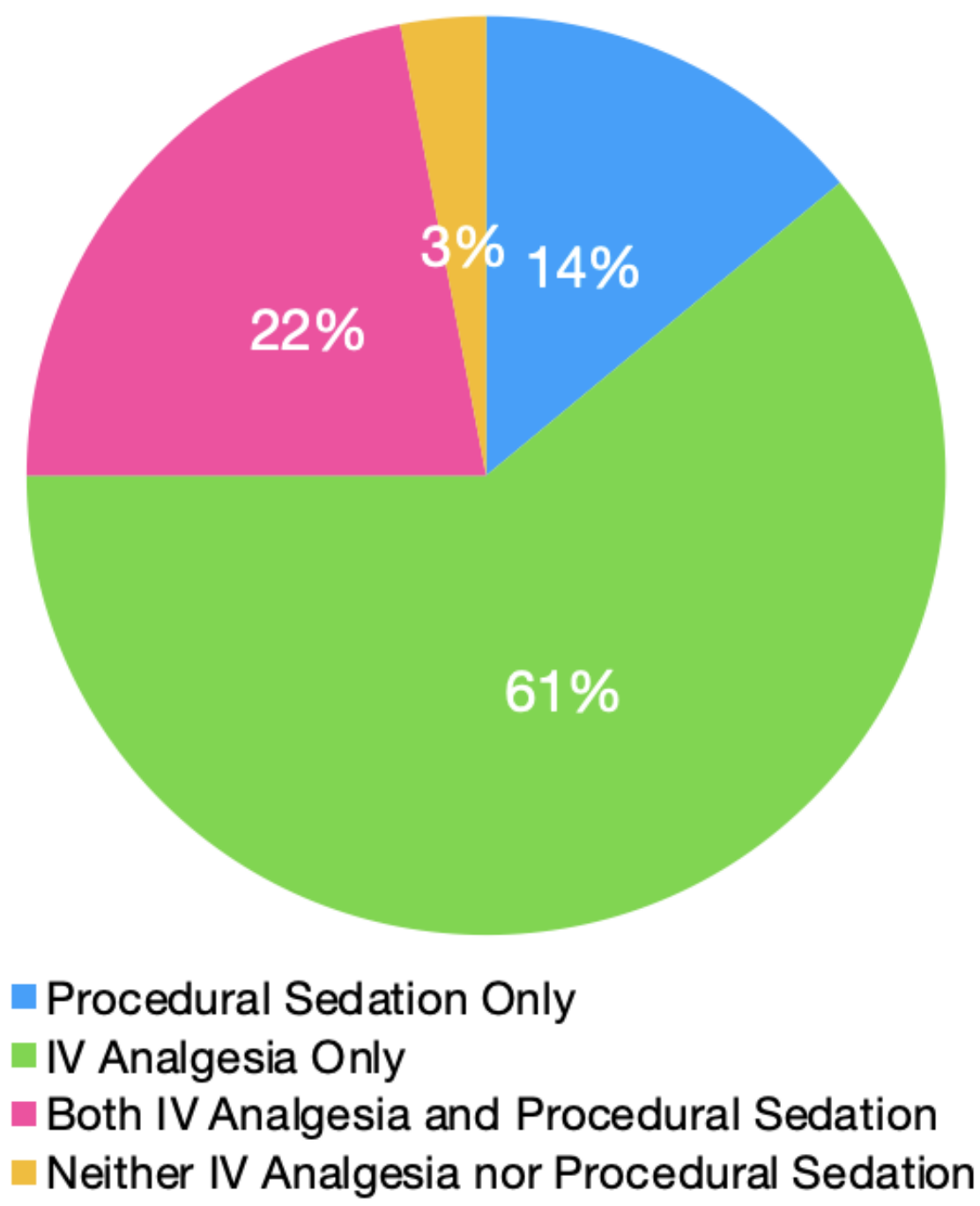
3%
14%
22%
61%
Procedural Sedation Only
IV Analgesia Only
Both IV Analgesia and Procedural Sedation
Neither IV Analgesia nor Procedural Sedation
